# Structural basis for the transition from translation initiation to elongation by an 80S-eIF5B complex

**DOI:** 10.1038/s41467-020-18829-3

**Published:** 2020-10-06

**Authors:** Jinfan Wang, Jing Wang, Byung-Sik Shin, Joo-Ran Kim, Thomas E. Dever, Joseph D. Puglisi, Israel S. Fernández

**Affiliations:** 1 Department of Structural Biology, Stanford University School of Medicine, Stanford, CA USA; 2 Department of Biochemistry and Molecular Biophysics, Columbia University, New York City, NY USA; 3 Eunice Kennedy Shriver National Institute of Child Health and Human Development, NIH, Bethesda, MD USA

**Keywords:** RNA, Ribosome, tRNAs, Cryoelectron microscopy

## Abstract

Recognition of a start codon by the initiator aminoacyl-tRNA determines the reading frame of messenger RNA (mRNA) translation by the ribosome. In eukaryotes, the GTPase eIF5B collaborates in the correct positioning of the initiator Met-tRNA_i_^Met^ on the ribosome in the later stages of translation initiation, gating entrance into elongation. Leveraging the long residence time of eIF5B on the ribosome recently identified by single-molecule fluorescence measurements, we determine the cryoEM structure of the naturally long-lived ribosome complex with eIF5B and Met-tRNA_i_^Met^ immediately before transition into elongation. The structure uncovers an unexpected, eukaryotic specific and dynamic fidelity checkpoint implemented by eIF5B in concert with components of the large ribosomal subunit.

## Abstract (web summary)

The GTPase eIF5B is involved in the correct positioning of the initiator Met-tRNA_i_^Me^^t^ on the ribosome during the late stages of translation initiation. Here the authors present a cryo-EM structure of the ribosome in complex with eIF5B and Met-tRNA_i_^Met^ immediately before transition into elongation, providing insight on Met-tRNA_i_^Met^delivery and how the correct reading frame is established.

## Introduction

**Fig. 1:**
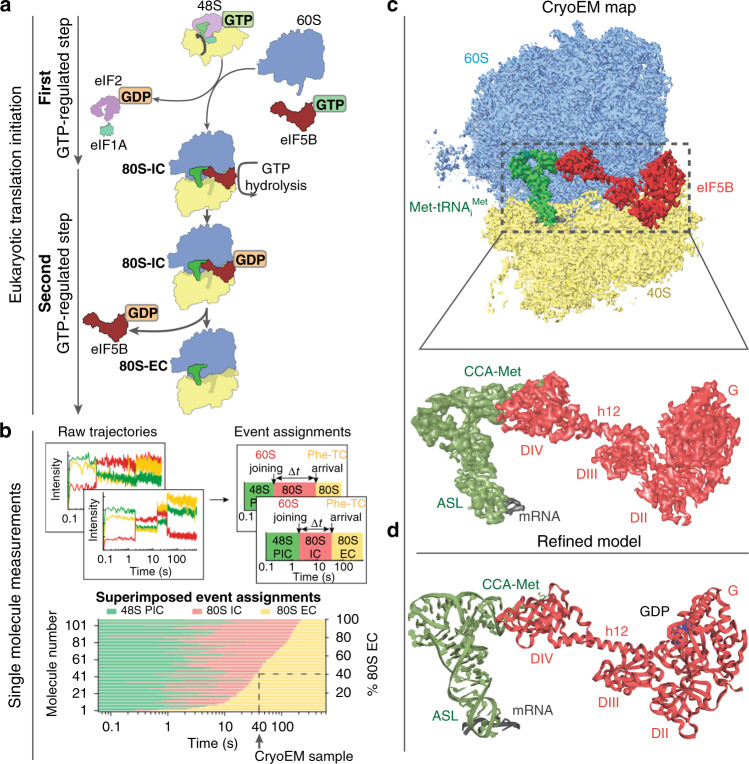
Cryo-EM sample preparation guided by single-molecule fluorescence data and overall architecture. **a** Overview of translation initiation in eukaryotes. Two steps, regulated by GTPases eIF2 and eIF5B control the delivery of Met-tRNA_i_^Met^. **b** Cryo-EM samples were prepared and froze at the timepoint 40 s after mixing 48S PICs, eIF5B:GTP, and 60S. At this timepoint, ~40% of the total 80S particles are expected to be 80S EC, while the other ~60% are 80S IC with eIF5B-bound, as demonstrated by our previous single-molecule fluorescence data (Supplementary Fig. 1)^11^. **c** Overview of the final cryo-EM map obtained after maximum likelihood classifications in Relion^44^. Large subunit (60S) is colored in blue, small subunit (40S) yellow, Met-tRNA_i_^Met^ green, mRNA gray, and eIF5B red. Bottom, details of the local map obtained for Met-tRNA_i_^Met^, mRNA, and eIF5B. Domain IV (DIV) of eIF5B is in close contact with the acceptor stem of Met-tRNA_i_^Met^. **d** Stereochemically refined models for Met-tRNA_i_^Met^, mRNA, and eIF5B with components indicated.

Protein synthesis starts with the assembly of a ribosomal complex at the start site on the mRNA^1^. Eukaryotes employ numerous translation initiation factors (eIFs) to achieve the 80S initiation complex (IC) with the initiator aminoacyl-tRNA (Met-tRNA_i_^Met^) and the AUG start codon of the mRNA programmed in the ribosomal peptidyl-tRNA site (P site)^2^. The 40S small ribosomal subunit, accompanied by eIFs and Met-tRNA_i_^Met^, is recruited to the 5′-untranslated region (5′-UTR) of the 7-methylguanosine-capped mRNA^3^. This is followed by a dynamic inspection of the 5′-UTR in search of the correct AUG codon as a start site, a process termed “scanning”^4,5^. Upon AUG recognition by the Met-tRNA_i_^Met^ anticodon, a series of eIF reorganizations and departures coupled to ribosomal conformational rearrangements occurs, resulting in a post-scanning 48S preinitiation complex (PIC)^6–8^. This 48S PIC is then joined by the 60S large ribosomal subunit to form the 80S IC, catalyzed by the universally conserved GTPase eIF5B (Fig. 1a)^9,10^. Using single-molecule fluorescence methods, we recently revealed that progression of the *Saccharomyces cerevisiae* 80S IC to the elongation-competent state (80S EC) with an exposed codon in the A site for aminoacyl-tRNA delivery is gated by the slow eIF5B dissociation from the complex (Supplementary Fig. 1a, b)^11^. This dissociation of eIF5B requires GTP hydrolysis, with the timing of its dissociation critical to stringent start-codon selection.

These dynamics results left unanswered whether the slow dissociation of eIF5B was limited by its GTPase activation/GTP hydrolysis, Pi release, and/or eIF5B:GDP dissociation. Previous cryo-EM reconstructions have described the architectures of the 80S IC bound with eIF5B:GDPCP at medium resolution, representing snapshots of the assembly prior to eIF5B GTP hydrolysis^12,13^. In addition, Met-tRNA_i_^Met^ has sequence features that allow it to bind directly to the P site of the ribosome during initiation, unlike elongator tRNAs^14–16^. Here, guided by the kinetics determined by our previous single-molecule fluorescence measurements, we performed cryo-EM analysis of the on-pathway initiation complexes to provide high-resolution information on the molecular mechanism by which eIF5B assists on the positioning of the initiator tRNA into the ribosomal P site and gates the transition from initiation to elongation.

## Results and discussion

### Architecture of the eIF5B-bound 80S IC

Leveraging the slow dissociation of eIF5B from the 80S IC in the native initiation pathway (average lifetime of the eIF5B-bound 80S state is 30–60 s at 20 °C)^11^, we prepared and froze samples at a pre-steady-state reaction timepoint (40 s, Fig. 1b, Supplementary Fig. 1, and “Methods”) corresponding to when ~60% of the assembled 80S should be bound with eIF5B (80S IC) after mixing 48S PICs with eIF5B:GTP and 60S subunits. Image processing and 3D classification in Relion3^17,18^ identified a homogeneous class of ~70% of the total pool of 80S particles, allowing the reconstruction of a 3D map with a global resolution of 2.9 Å (Supplementary Figs. 2–4). The reconstruction shows clear density for a tRNA in the P-site region and density for all domains of eIF5B (Fig. 1c). Further analysis of the map revealed excellent densities for domain III (DIII), the connecting α-helix 12 (Fig. 1c, d, h12^19^) and especially for domain IV (DIV) of eIF5B, as well as for the Met-tRNA_i_^Met^ and the start codon of the mRNA (Fig. 1c, d, and Supplementary Fig. 4). Less well-resolved were domains II and G of eIF5B and the elbow region of Met-tRNA_i_^Met^ (Supplementary Fig. 3).

### eIF5B DIV stabilizes the acceptor stem of Met-tRNA_i_^Met^ near the PTC

**Fig. 2:**
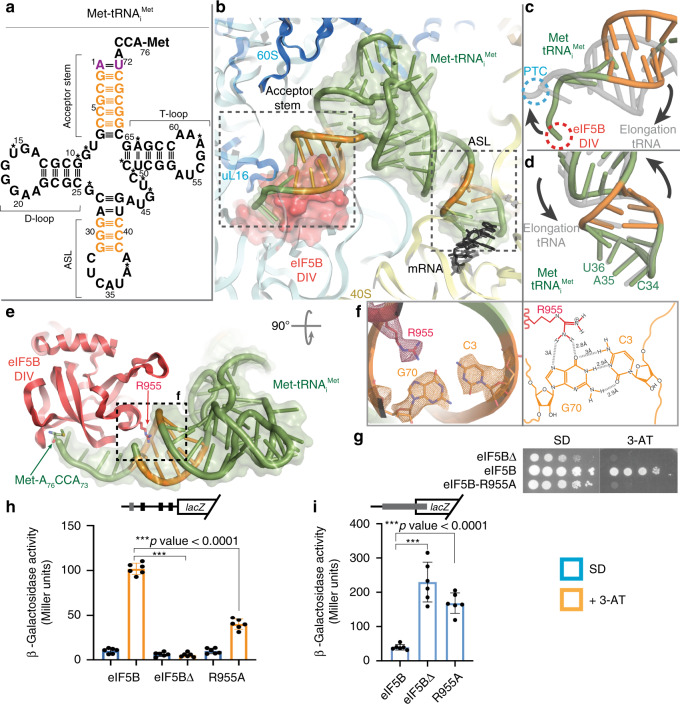
Role of the G–C base-pairs clusters in Met-tRNA_i_^Met^ conformation. **a** Sequence diagram of the *S. cerevisiae* Met-tRNA_i_^Met^ with initiator-specific base pairs colored in orange and modified nucleotides marked with asterisks. **b** Overview of the conformation adopted by Met-tRNA_i_^Met^ on the 80S-IC complex with the initiator-specific G–C base pairs colored in orange. **c** Superposition of Met-tRNA_i_^Met^ (green) with a P site classical-state elongation tRNA (gray) reveals a distorted configuration of the acceptor stem of Met-tRNA_i_^Met^ when compared with an elongation peptidyl-tRNA. This distortion prevents the _74_CCA_76_-Met end of Met-tRNA_i_^Met^ from reaching the peptidyl transferase center (PTC, blue) on the 60S. **d** In contrast, the ASL of Met-tRNA_i_^Met^ has reached its final position when compared with an elongation tRNA in the P site. **e** The arginine residue 955 at DIV of eIF5B specifically recognizes the Hoogsteen edge of the base of nucleotide G70 of Met-tRNA_i_^Met^ (red arrow, R955). **f** Cryo-EM density for side chains and individual bases in the area around eIF5B R955. On the right, chemical diagram with contacts and distances indicated. **g**–**i** Yeast growth and *GCN4-lacZ* reporter assays of strains lacking eIF5B or expressing wild-type or R955A mutant. Cells were spotted on minimal (SD) or starvation (3-AT) medium (**g**) or assayed for ß-galactosidase activity following introduction of a wild-type *GCN4-laZ* reporter (**h**) or a derivative in which an extended version of uORF1 overlaps, out-of-frame, the *GCN4* start codon (**i**). Results are the average and standard deviation of six independent transformants combined from two independent experiments with three replicates each; *p* values were calculated using two-sided Student’s *t* test. The *p* values for panel (**h**) are eIF5B vs. eIF5BΔ: 7.57349 × 10^−12^, eIF5B vs. eIF5B-R955A: 6.30158 × 10^−9^ and for panel (**i**) are eIF5B vs. eIF5BΔ: 1.23215 × 10^−5^, eIF5B vs. eIF5B-R955A: 1.3313 × 10^−6^.

In our reconstruction, DIV of eIF5B is tightly packed against the peptidyl transferase center (PTC) of the large subunit where it contacts the _73_ACCA_76_-Met end of Met-tRNA_i_^Met^ (Fig. 2). The close interaction of eIF5B DIV with the acceptor stem of Met-tRNA_i_^Met^ enforces a change in the trajectory of the _73_ACCA_76_-Met end when compared with the position adopted by the acceptor stem on an elongation peptidyl-tRNA in a canonical configuration (Supplementary Movie 1)^20^. This distortion is not limited to the _73_ACCA_76_-Met of tRNA_i_^Met^, as the whole acceptor stem is distorted compared with its position in an elongation tRNA (Fig. 2b, c). The cluster of G–C base pairs in the acceptor stem specific to Met-tRNA_i_^Met^ seems to play a pivotal role in this context, allowing a specific distortion of this stem as the Met-tRNA_i_^Met^ simultaneously interacts with eIF5B and the start codon (Fig. 2a, b, and Supplementary Fig. 5). Basic residues of the domain IV of eIF5B make specific interactions with this G–C base-pairs cluster^21^; Arg955 of eIF5B contacts the base of G70 of Met-tRNA_i_^Met^ from the major groove of the acceptor stem (Fig. 2e, f). Although mutating Arg955 to Ala in eIF5B did not substantially alter the growth rate of yeast in rich medium, the mutant strain could not grow under amino acid starvation conditions (Fig. 2g), suggesting impaired *GCN4* expression. The *GCN4* mRNA contains four upstream open reading frames. Translation of the first uORF (uORF1) enables regulated reinitiation at the subsequent uORFs to control *GCN4* expression. The Arg955 to Ala mutation in eIF5B significantly impaired derepression of *GCN4-lacZ* reporter expression under starvation conditions (Fig. 2h), consistent with the growth defect conferred by this mutation under these conditions. Moreover, using a reporter in which the leader was altered and uORF1 was extended to overlap and thus block *GCN4* expression (Fig. 2i), the mutation was found to increase the frequency of ribosomes scanning past the start codon (termed “leaky scanning”) of uORF1 and translating *GCN4*. Thus, the interaction between eIF5B Arg955 and Met-tRNA_i_^Met^ G70 may also play an important role in start-site selection, and the enhanced leaky scanning of the stimulatory uORF1 might partially account for the defect in *GCN4* expression. Consistently, deletion of eIF5B DIV also enhanced leaky scanning and blocked *GCN4* expression in yeast^19^ (Fig. 2h, i).

### A eukaryotic specific Met-tRNA_i_^Met^ recognition mechanism

**Fig. 3:**
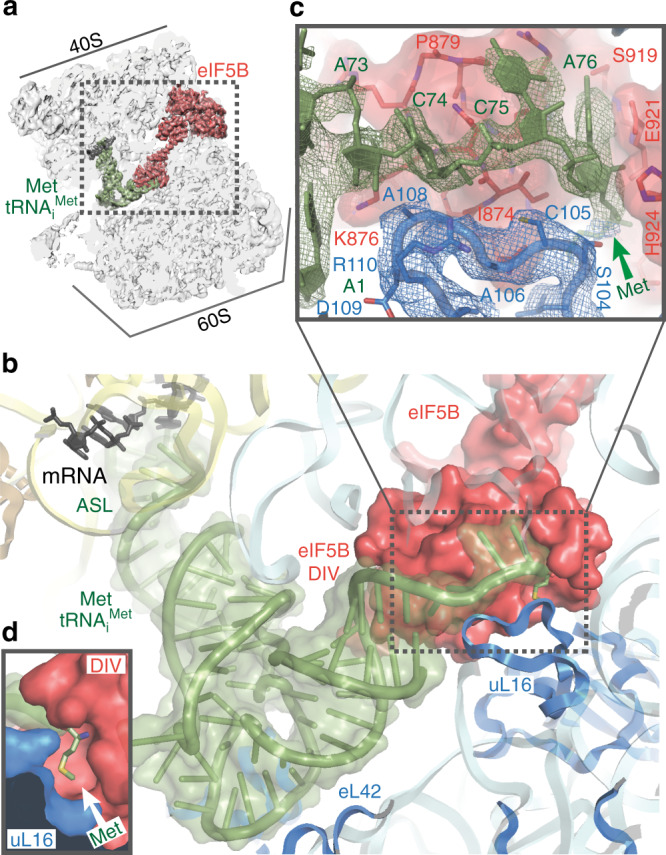
Ribosomal protein uL16 collaborates with eIF5B DIV in Met-tRNA_i_^Met^ A76-Met recognition. **a** Overall view of the 80S/Met-tRNA_i_^Met^/eIF5B complex in an orientation centered on eIF5B DIV. **b** A detailed view of the Met-tRNA_i_^Met^ conformation adopted while simultaneously interacts with the mRNA and eIF5B DIV on the 80S. **c** A detailed view of eIF5B DIV focused on the acceptor stem region of Met-tRNA_i_^Met^. DIV of eIF5B is shown in red as semi-transparent Van der Waals surface, Met-tRNA_i_^Met^ nucleotides depicted in green with experimental cryo-EM density shown and uL16 residues are in blue with experimental cryo-EM density shown. **d** DIV of eIF5B and residues 102–110 of uL16 form a hydrophobic cavity where the methionine residue of Met-tRNA_i_^Met^ is hosted.

The high quality of the map around the PTC region revealed specific contacts of residues of eIF5B DIV with the four-terminal bases of the Met-tRNA_i_^Met^ as well as with the ribose and phosphate backbone atoms (Fig. 3). Specifically, the base of Met-tRNA_i_^Met^ nucleotide A76 is tightly monitored by eIF5B residues Glu921 and His924, which anchors the adenine moiety to eIF5B DIV (Fig. 3b, c). In this orientation, the methionyl group esterified to the 3′OH of the A76 ribose is directed towards a hydrophobic “pocket” formed by the surface of eIF5B around residue Ile874 (Fig. 3c, d, and Supplementary Fig. 4c).

Intriguingly, this “hydrophobic pocket” is capped by a loop of ribosomal protein uL16, a component of the 60S (Fig. 3d), which is conserved in yeast and humans (Supplementary Fig. 6). This uL16 loop, formed by residues 100 to 120, is disordered in reported elongation 80S complexes, but is well-resolved in our reconstruction, allowing its modeling and refinement. Notably, this loop is stringently checked at the last step of 60S biogenesis by sophisticated cellular machinery that blocks 60S exporting to the cytoplasm if its integrity is compromised^22^. No clear function has been assigned for this loop that would justify such a conserved and costly cellular machinery^23^.

**Fig. 4:**
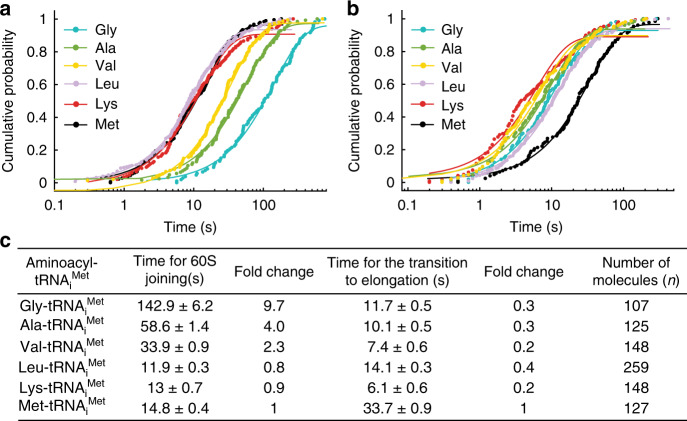
Kinetics of 60S subunit joining and the subsequent transition into elongation in experiments performed with mis-acylated tRNA_i_^Met^. The cumulative probability distributions of the dwell times for 60S joining (**a**) and the transition to elongation (**b**) from experiments performed at 3 mM free Mg^2+^ and 20 °C with the model mRNA and unlabeled eIF5B. Data points were fitted to a single-exponential equation with the mean times reported in (**c**). Errors represent 95% confidence intervals, and the number of molecules analyzed (*n*) are listed in (**c**).

Here, residues Ser104 to Arg110 of this loop are located in close proximity to the _73_ACCA_76_-Met of Met-tRNA_i_^Met^, in an almost parallel configuration to the phosphate backbone of the Met-tRNA_i_^Met^ (Fig. 3c). In addition, uL16 residues Leu103 and Ser104 tightly approach the eIF5B residues around Leu871 to define a narrow and hydrophobic cavity where the methionine residue is hosted (Fig. 3d and Supplementary Fig. 6). No specific contacts between the methionine moiety and either eIF5B or uL16 residues could be observed, which suggests that the recognition of the amino acid attached to an initiator tRNA is not achieved via a specific interaction with eIF5B or uL16 residues. To explore the determinants that govern such recognition mechanism, we systematically mis-acylated tRNA_i_^Met^ with five different amino acids with increasing side chain volumes and hydrophobicity or introducing a positive charge (Fig. 4)^24^.

All the mis-acylated tRNAs are active in 48S PIC and 80S formation (Fig. 4). However, the 60S subunit joining rate is altered in a way that reflects a predilection for larger side chains: the stepwise increase of the side chain size from Gly to Ala, Val, Leu, and even the positively charged Lys resulted in increasing 60S joining rates, progressively approaching the values observed for Met (Fig. 4a, c). In addition, the mean times for the transition to elongation all decreased substantially to a similar level (by ~3–5-fold) due to the mis-acylation. Both the altered 60S arrival times and faster transitions into elongation might reflect weakened interactions between the aminoacyl-tRNA_i_^Met^ and eIF5B/uL16 when the methionine moiety was replaced by other amino acids, reflecting the importance of the size of the side chain esterified to A76 of tRNA_i_^Met^. The cavity formed by eIF5B DIV and the uL16 loop (residues 100–120) could thus act as a selectivity “filter” regarding the identity of the amino acid.

### AUG recognition by the Met-tRNA_i_^Met^ anticodon

**Fig. 5:**
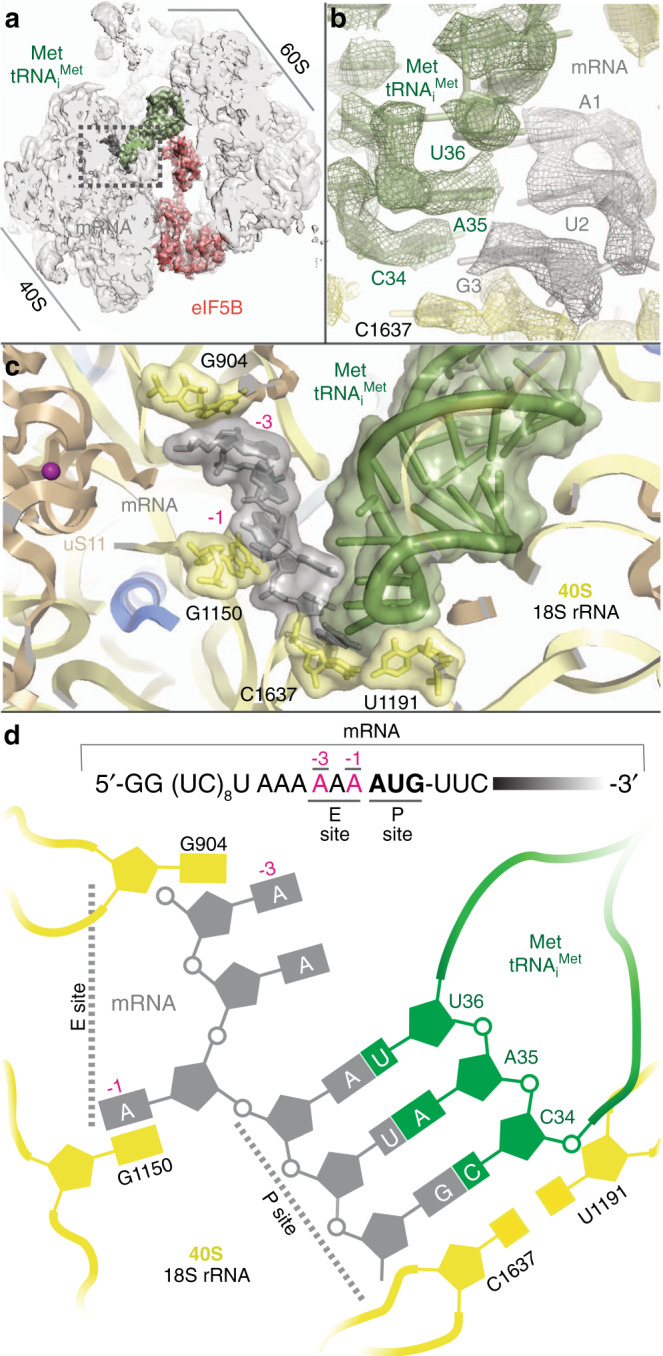
mRNA nucleotides at positions −1/−3 play a key role in later stages of initiation. **a** Overall view of the 80S/Met-tRNA_i_^Met^/eIF5B complex in an orientation centered on the mRNA. **b** Detailed view of the small ribosomal subunit P site with experimental cryo-EM density shown. The Met-tRNA_i_^Met^ ASL is in green, the start codon gray, and ribosomal bases of the small subunit yellow. **c** Overall view of the P and E sites of the small ribosomal subunit. Six bases corresponding to the start codon of the mRNA and three preceding bases corresponding to nucleotides −1 to −3 showed unambiguous densities and could be modeled (gray). The final refined model with labeled components is shown. **d** Simplified schematic view of the conformation adopted by the start codon in the P site and the preceding −1 to −3 bases in the E site in the presence of eIF5B.

The anticodon arm of Met-tRNA_i_^Met^ features a configuration very similar to that described for a peptidyl-tRNA in an elongation, canonical state (Fig. 5, Supplementary Fig. 5, and Supplementary Movie 1)^20^. Anticodon bases C34, A35, and U36 have reached their final elongation position, which would allow a productive transition into elongation. The G–C cluster of base pairs in the ASL seems to play an important role, allowing a local distortion that guarantees an ideal positioning of the anticodon bases to maximize the interaction with the AUG codon^15^, while allowing the tRNA to bend at the elbow region of T-loop/D-loop tertiary interaction to prevent accommodation of the aminoacyl-acceptor stem to the elongation state (Fig. 2b–d). In addition, the A minor type interactions between 40S residues at the P site and the Met-tRNA_i_^Met^ ASL at the G–C base-pair cluster are similar to those described to be important in bacterial initiation, highlighting the functional importance of the G–C base-pair cluster in the ASL of tRNA_i_^Met^ throughout evolution (Supplementary Fig. 5c)^25^. Thus, both G–C base-pair clusters at the acceptor stem and the anticodon arm of Met-tRNA_i_^Met^ are essential to allow a specific conformation of the Met-tRNA_i_^Met^ on the P site in both the early^21^ and late stages of initiation^14^.

For the mRNA, we could unambiguously identify six nucleotides including the AUG start codon and the three nucleotides immediately upstream (Fig. 5). Only weak densities ascribable to the A-site codon could be observed, and no ordered RNA density could be identified at the entry nor exit sites of the mRNA channel on the 40S. This is in marked contrast with 48S PIC structures from earlier initiation stages, in which long stretches of the mRNA from the entry to the exit sites were well resolved^5^, pinpointing a less prominent role of mRNA/40S interactions once initiation has progressed into its later stages.

The codon-anticodon interaction observed in our reconstruction is virtually identical to a canonical cognate pair (Fig. 5b, c)^20^. Nucleotides C1637 and U1191 of the 18S rRNA at the base of the 40S P-site bracket the base pair mRNA-_3_G:C_34_-tRNA_i_^Met^ in a very similar configuration as in an elongation complex (Fig. 5c, d). In addition, ribosomal bases G1150 and G904 engage mRNA nucleotides at position −1 and −3, respectively, in stacking interactions in a similar configuration as in early initiation complexes in the 48S PIC context (Fig. 5c, d)^6,7^. Thus, in the later stages of initiation, prior to entrance into elongation, the start codon and its flanking residues present a hybrid configuration with bases at position −1/−3 retaining key contacts with rRNA bases that are instrumental in the early positioning of the mRNA on the 40S and, at the same time, with the start-codon features characteristic of a standard conformation of an elongation state^20^. Hence, the start codon-surrounding sequence, especially the bases at positions −3/−1, plays an essential role along all initiation, from early “scanning” to later entrance into elongation^26^.

### Release of eIF5B-GDP limits the rate of the transition into elongation

Analysis of the G domain of eIF5B that binds GTP reveals clear density for a bound nucleotide that, however, lacks features compatible with the presence of a γ-phosphate, implying that the nucleotide state is either GDP or GDP-Pi (Supplementary Fig. 4d)^27,28^. The absence of density ascribable to the γ-phosphate even at a low threshold (Supplementary Fig. 4d, right) points towards a nucleotide state with GDP. This is additionally supported by the fact that the switch I of eIF5B is disordered, which differs from the ordered state prior to GTP hydrolysis^27,29^. Thus, our reconstruction represents an intermediate of the 80S IC right before its transition to the elongation-competent state, but post GTP hydrolysis. This is further supported by the fact that the ribosomal inter-subunit configuration observed here is different from the 80S IC state prior to GTP hydrolysis (Supplementary Fig. 7). A ~3° counterclockwise rotation of the 40S in relation to the 60S was observed in the pre-GTP hydrolysis state, which was coupled with apparent 40S head swivel (Supplementary Fig. 7^12,13,30^). However, the eIF5B-bound 80S IC observed here presents a configuration very similar to a canonical non-rotated 80S complex, with virtually no rotation of the small subunit and minimal swiveling of the 40S head (Supplementary Fig. 7). Thus, the 80S IC will reconfigure its conformation after GTP hydrolysis to a state that is more similar to the non-rotated elongation-competent state, and this reconfiguration is coupled to the structural rearrangements of eIF5B.

**Fig. 6:**
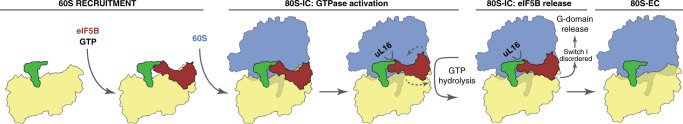
A structural view of the on-ribosome eIF5B catalysis. After Met-tRNA_i_^Met^ (green) is delivered to the P site of the small subunit (40S, yellow) and the start codon is recognized, eIF5B in its GTP-bound form is able to transiently stabilize the aminoacyl-tRNA in the P site and catalyze the recruitment of the large subunit (60S, blue). Proper positioning of Met-tRNA_i_^Met^, eIF5B, and the 60S would lead to the structuring of the uL16 loop (residues 100–120) which in turn anchors eIF5B DIV solidly to the 60S in the vicinity of the PTC. The successful establishment of these interactions signals for eIF5B GTPase activation and GTP hydrolysis, which happens in the G domain of eIF5B placed near the GTPase activation center of the 60S. GTP hydrolysis by eIF5B is followed by the de-structuring of switch I, which reduces the affinity of the factor to the ribosome. The final departure of eIF5B from the ribosome is likely implemented in two steps, beginning with the super-domain G/II and followed by the detachment of DIV-h12-DIII.

In summary, our structural analysis of the pre-steady-state initiation complexes identified a novel intermediate of the late translation initiation complex on the native reaction pathway. Our results demonstrated that GTP hydrolysis and Pi release are not the rate-limiting steps for eIF5B dissociation from the 80S IC, but the subsequent eIF5B-GDP dissociation. Notably, our structure describes stable interactions among Met-tRNA_i_^Met^, eIF5B DIV, and uL16 after GTP hydrolysis by eIF5B (Fig. 6). Disruption of this network of contacts is required for release of eIF5B, perhaps explaining the slow eIF5B departure rate post GTP hydrolysis. Such cooperation among Met-tRNA_i_^Met^, an initiation factor and the 60S subunit highlights a eukaryote-specific mechanism to control the progression of the initiation complex into the elongation phase.

## Methods

### Materials

All the yeast *Saccharomyces cerevisiae* 40S and 60S ribosomal subunits, initiation factors eIF1, 1A, 2, 5, and 5B (residues 396–1002), and mRNA were prepared and characterized as described^11^. The model mRNA, with the sequence: GG(UC)_8_UAAAAAAAUGUUCAAAUAA(UC)_16_, was an uncapped, unstructured model mRNA containing the optimal yeast 5′ context consensus sequence (underlined) with a biotin covalently linked to the 3′end^11^. Native yeast methionylated initiator tRNA (Met-tRNA_i_^Met^) was purchased from tRNA Probes, LLC (MI-60).

### 80S:eIF5B complex assembly on model mRNA

Previously, by applying single-molecule fluorescence microscopy methods to a purified, reconstituted yeast translation system, we have revealed that eIF5B is the gating factor during the transition from eukaryotic translation initiation to elongation^11^. We preformed post-scanning 48S preinitiation complexes (48S PICs, wherein 40S was labeled with a Cy3 dye) which were immobilized on zero-mode waveguides (ZMWs) imaging surface via a biotin at the 3′end of the mRNAs (Supplementary Fig. 1a)^11^. After washing away unbound components, 60S (Cy5-labeled), eIF5B (Cy5.5-labeled), and the first elongator Phe-tRNA^Phe^ (Cy3.5-labeled) in ternary complex with the elongation factor eEF1A and GTP (Phe-TC) were delivered along with required eIFs to start the reaction. By directly illuminating all the fluorescent dyes, we could monitor, in real time, the order of molecular events occurring during the late translation initiation stages and its transition to elongation (Supplementary Fig. 1b)^11^. This allowed us to measure and determine the occupancy times of eIF5B on newly formed 80S complexes on the native reaction pathway. In particular, for the model mRNA, the mean time of 60S joining to form an 80S was ~16 s with the eIF5B occupancy time on the 80S ~34 s at 20 °C and 3 mM free Mg^2+^; whereas these values were ~3.6 s and 68.7 s at 20 °C and 10 mM free Mg^2+^, respectively^11^. Simulating the kinetic curves from these two reactions provided the information about the time-evolution of the populations of different complexes (Supplementary Fig. 1c)^11^. To aid the reconstruction of a high-resolution structure of the on-pathway eIF5B-bound 80S prior to its transition to elongation without the need of non-hydrolysable GTP analogs or mutants, we decided to prepare and freeze our sample at timepoint 40 s at 20 °C and 10 mM free Mg^2+^, where we would expect the eIF5B-bound 80S population accounts ~60% of the total 80S particles (Supplementary Fig. 1c).

Samples were prepared in the buffer containing 30 mM HEPES-KOH pH 7.5, 100 mM KOAc, 10 mM Mg(OAc)_2_, and 1 mM GTP:Mg^2+^. First, a ternary complex mixture was prepared by pre-incubating 3.8 μM eIF2 at 30 °C for 10 min, followed by another 5 min incubation at 30 °C after addition of 2.8 μM Met-tRNA_i_. Next, this ternary complex mixture was diluted to one-third of the concentration, and incubated at 30 °C together with 1 μM eIF1, 1 μM eIF1A, 0.5 μM model mRNA, and 0.3 μM 40S for 15 min, resulting a 48S PIC mixture. Separately, a 60S mixture was prepared by mixing 1 μM eIF5, 1 μM eIF5B, and 0.15 μM 60S. The 48S PIC and 60S mixtures were kept on ice before sample freezing. In parallel, the 200-mesh Quantifoil R2/1 grids (Electron Microscopy Sciences, Q250AR1) were glow-discharged for 25 s in a PELCO EasiGlow glow discharger (Ted Pella, Inc., conditions: negative charge, 15 mA, 0.4 mBar, 25 s). After prewarming the samples to room temperature, 3.5 μL of the 48S PIC mixture was mixed with 3.5 μL of the 60S mixture. A 3 μL sample from the resulting mixture was applied to each grid at 21 °C and 95% humidity. The sample was vitrified by plunging into liquid ethane after 2.5 s blotting using a Leica EM GP (Leica Microsystems) plunger. The total time from combining the 48S PIC and 60S mixtures to grid freezing was ~40 s.

### Single-molecule experiments with mis-acylated tRNA_i_^Met^

Native yeast tRNA_i_^Met^ was mis-acylated by the flexizyme dFx with Gly-, Ala-, Val-, Leu-, and Lys-DBE as described^24,31^. Real-time single-molecule experiments on the ZMW-based PacBio RSII instrumentation and data analyses were performed with exactly the same methodology as previously described^11^. The mean times of 60S arrival to the immobilized 48S PICs (60S arrival time) and the subsequent transition to elongation were determined in experiments performed with the model mRNA and unlabeled eIF5B at 3 mM free Mg^2+^ and 20 °C.

### Generation of eIF5B mutant and growth and reporter experiments in yeast

The *Saccharomyces cerevisiae fun12Δ* strain J111 (*MATa ura3-52 leu2-3 leu2-112 fun12Δ*)^32^ lacking eIF5B was transformed with an empty vector (eIF5BΔ) or plasmids expressing N-terminally deleted (lacking residues 28–396) form of wild-type (WT) eIF5B^32^ or the eIF5B-R955A mutant, as indicated. Transformants were grown to saturation, and 5 µl of serial dilutions (of OD_600nm_ = 1.0, 0.1, 0.01, 0.001, and 0.0001) were spotted on minimal medium supplemented with essential nutrients (SD) or medium containing 10 mM 3-aminotriazole (3-AT) to cause histidine starvation. Plates were incubated for 3 days at 30 °C. For reporter assays, *wild-type GCN4-lacZ* plasmid p180^33^ or a deletion derivative in which uORF1 was moved to the position of uORF4 and then extended to overlap the *GCN4* AUG start codon (pM226)^34^ were introduced into the strains. Cells were grown under repressing, non-starvation, conditions in SD medium for 2 h, and then incubated under repressing or derepressing, starvation, conditions (SD + 10 mM 3-AT) for an additional 6 h; cells were harvested and ß-galactosidase activities were determined as described previously^34,35^.

### Data collection, image processing, and structure determination

Cryo-EM data was collected on a Titan Krios microscope (FEI) equipped with an energy filter (20 eV slit width) and a K3 direct detector (Gatan) operated at 300 KeV. A 70 µm C2 aperture was used with a pixel size of 0.83 Å/pixel and illumination conditions adjusted in nanoprobe mode to a fluence of 10e^−^/pixel/s. Four-second images with a frame width of 100 ms (1.45 e^−^/Å^2^/frame) were collected in counting mode. Contrast transfer function parameters were estimated using CTFIND4^36^. Particle picking was performed using Relion3.1^18^ without the use of templates and with the Laplacian module (max/min diameter 380/320 Å) identifying an initial set of particles. Eight times binned particles (extraction box 416 pixels) were subjected to an initial 2D classification job (200 classes, T parameter 2, 35 iterations) and those classes showing clear secondary structure features were selected for a 8xbin 3D auto-refine job. All 2D and 3D classifications and refinements were performed using RELION^17,18,37^.

Next, 3D classification without alignment and a mask including the inter-subunit space and the 40S head (four classes, T parameter 4) identified a class with unambiguous density for eIF5B and a tRNA in the P site^17^. This class was independently processed with unbinned data, yielding high-resolution maps with density features in agreement with the reported resolution after several cycles of ctf-refinement and a final step of Bayesian polishing. Local resolution was computed with RESMAP^38^.

### Model building and refinement

Models of yeast 40S, 60S^27^, Met-tRNA_i_^Met^, and eIF5B were docked into the maps using CHIMERA^39^, and COOT^40^ was used to manually adjust these initial models. An initial round of refinement was performed in Phenix using real-space refinement^41^ with secondary structure restraints and a final step of reciprocal-space refinement with REFMAC^42^. The fit of the model to the map over-fitting tests were performed following standard protocols in the field^43^.

### Reporting summary

Further information on research design is available in the Nature Research Reporting Summary linked to this article.

## Supplementary information

Supplementary Information

Reporting Summary

Description of Additional Supplementary Files

Supplementary Movie 1

## Data Availability

The data that support this study are available from the corresponding authors upon reasonable request. Cryo-EM maps have been deposited in the Electron Microscopy Data Bank under accession code EMD-21859 and coordinates at the Protein Data Bank under accession code 6WOO. Source data for Fig. 2h, i are included as Supplementary Table 2.

## Code availability

The MATLAB scripts used for single-molecule data analyses are available at https://drive.google.com/drive/u/1/folders/1-yKC0Nui7T7yoJScCuODPhCT4G52emLj.
