# Supplementary material for: Structural basis for the transition from translation initiation to elongation by an 80S-eIF5B complex
Source: Nat Commun. 2020 Oct 6;11:5003. doi: 10.1038/s41467-020-18829-3 (PMC7538418; doi:10.1038/s41467-020-18829-3)
Supplement: Supplementary file 1 — Supplementary Information [file 41467_2020_18829_MOESM1_ESM.pdf]

Supplementary Information

Structural basis for the transition from translation initiation to elongation by an 80S-eIF5B complex

Jinfan Wang, Jing Wang, Byung-Sik Shin, Joo-Ran Kim, Thomas E. Dever*, Joseph D. Puglisi* and Israel S. Fernández*
*Corresponding authors. Email: thomas.dever@nih.gov, puglisi@standford.edu, isf2106@cumc.columbia.edu

This PDF file includes:
Supplementary Fig. 1-7
Supplementary Movie 1
Supplementary Table 1 and 2
References

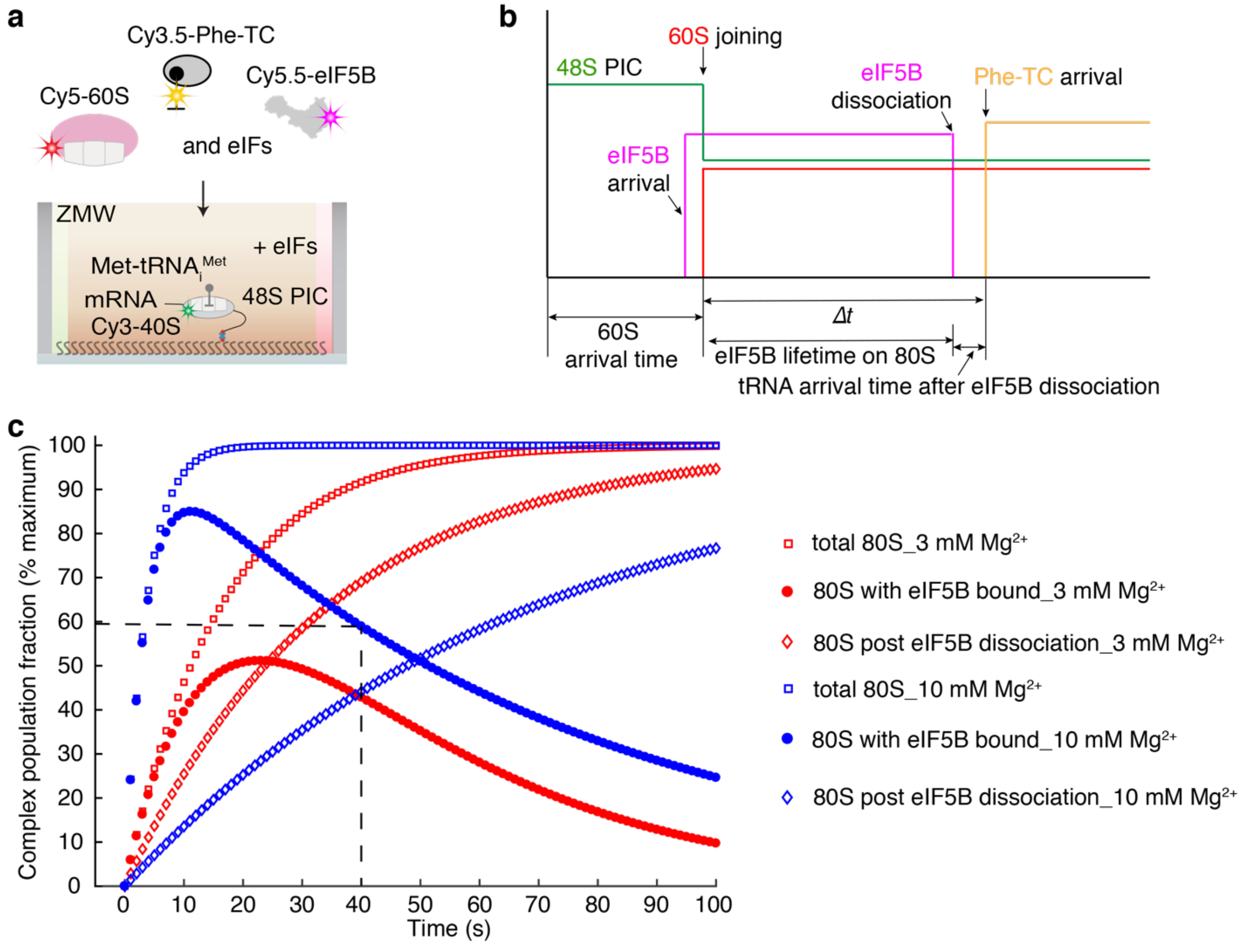

Supplementary Fig. 1. CryoEM sample preparation guided by single-molecule dynamics. Single-molecule fluorescence microscopy experimental set up (**a**) and representative schematic experimental trace (**b**) with the molecular events annotated[1]. Mean times for subunit joining to form the 80S IC (60S arrival time) and subsequent transition into elongation (Δt, signaled by the arrival of the first elongator Phe-TC) were estimated. Δt is obtained by the addition of the eIF5B lifetime on 80S and the tRNA arrival time after eIF5B dissociation, with the latter being much smaller than the former. (**c**) Simulated time-evolution of 80S complex populations based on the previously determined kinetics in experiments performed with the model mRNA at 20ºC[1]. Informed by our kinetics measurements, cryoEM samples corresponding to the timepoint 40 s after mixing 48S PICs, eIF5B:GTP and 60S in the presence of required eIFs at 10 mM free Mg^{2+} and 20ºC were frozen. Dashed line indicates the estimated timepoint when the 80S population with eIF5B is ~60%.

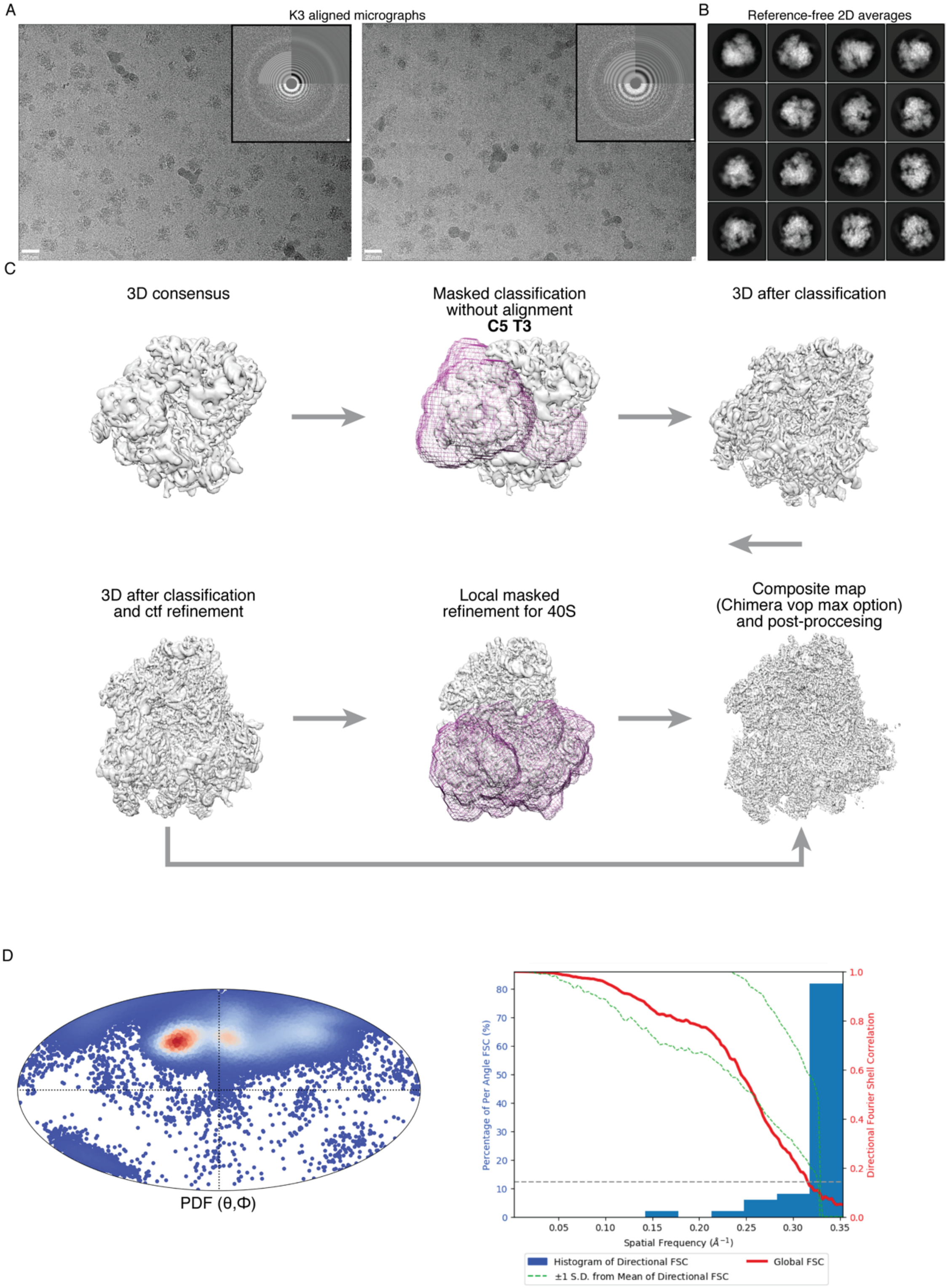

Supplementary Fig. 2. Representative cryoEM images, reference-free 2D averages and image processing workflow.

a Motion corrected micrographs. **b** Selected 2D averages. **c** Classification workflow. **d** On the left, Eulerian angles distribution of the final class and on the right directional FSC[2].

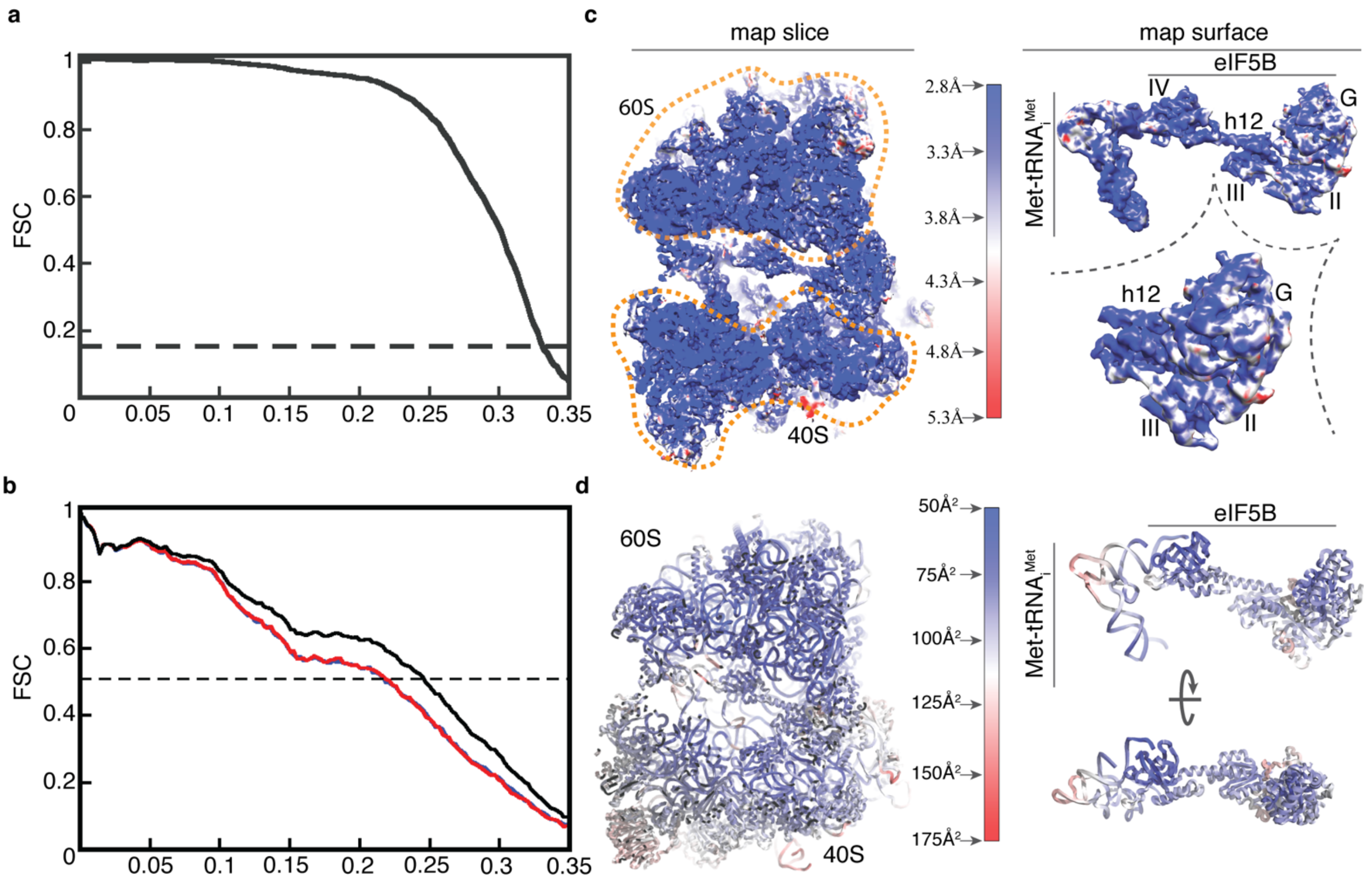

Supplementary Fig. 3. Fourier Shell Correlation curves, model validation and local resolution.

a Fourier Shell Correlation curve (FSC) for independently refined half-maps indicated a final resolution of 2.9Å. **b** Model validation FSC for the final "shacked" model and half-map 1 (blue line) and half-map 2 (red curve) not included in the refinement. Black line represents the FSC for the final model against the final, post-processed map used in model building and model refinement. The overlapping of the blue and red curve guarantees absence of overfitting in our model[3].**c** Unsharpened map colored according to local resolution calculations. On the right, a detailed view for the region of the map containing density for eIF5B and Met-$tRNA_i^{Met}$. **d** Final stereochemically refined model colored according to temperature B-factors estimated by REFMAC[4]. Regions of the model with higher B-factors correlate with flexible areas of the map.

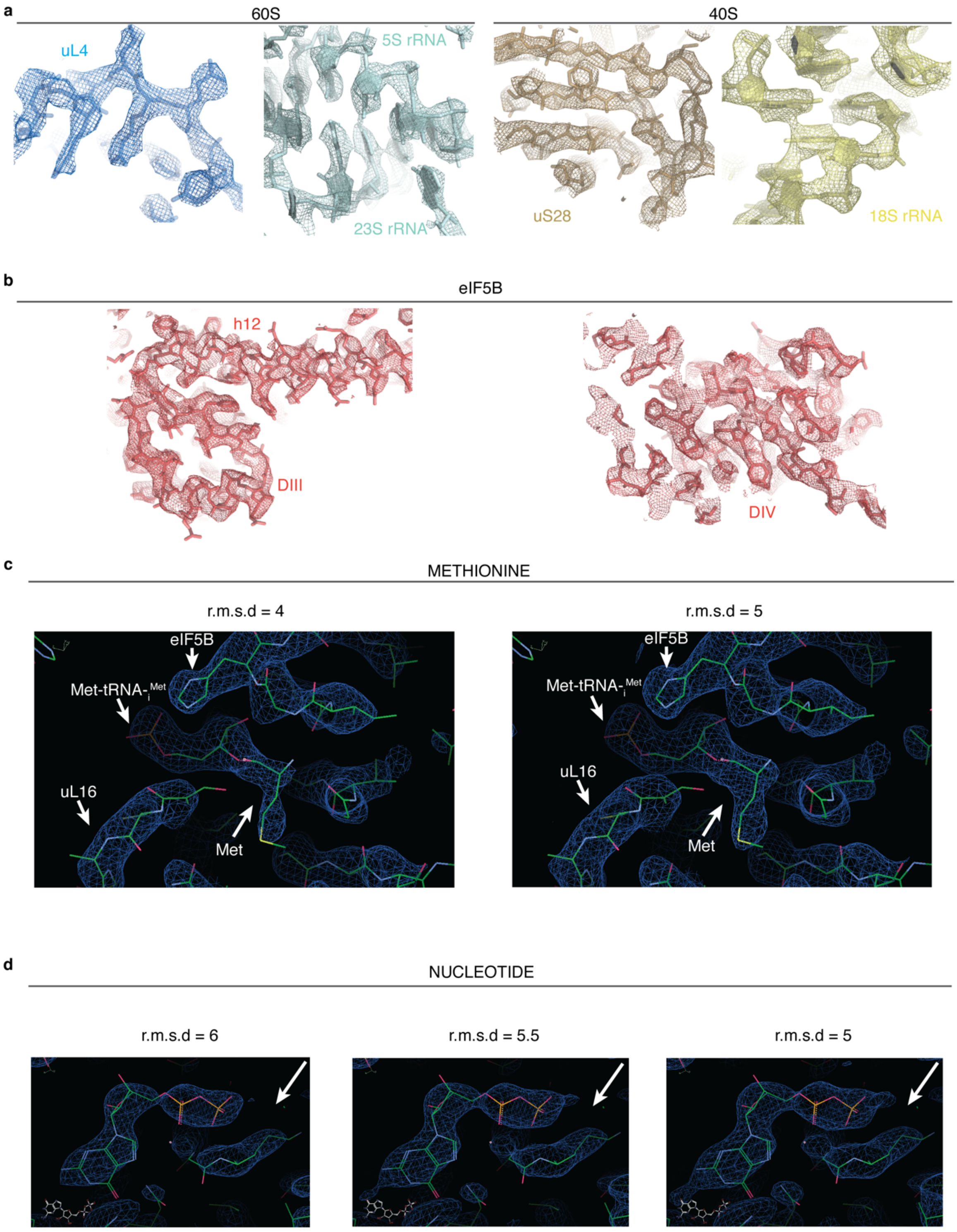

Supplementary Fig. 4. Representative cryoEM densities.
Final, post-processed cryoEM densities for: **a** 60S components and 40S components, **b** eIF5B, **c** Met-tRNA$_i^{Met}$, and **d** G domain and modelled GDP.

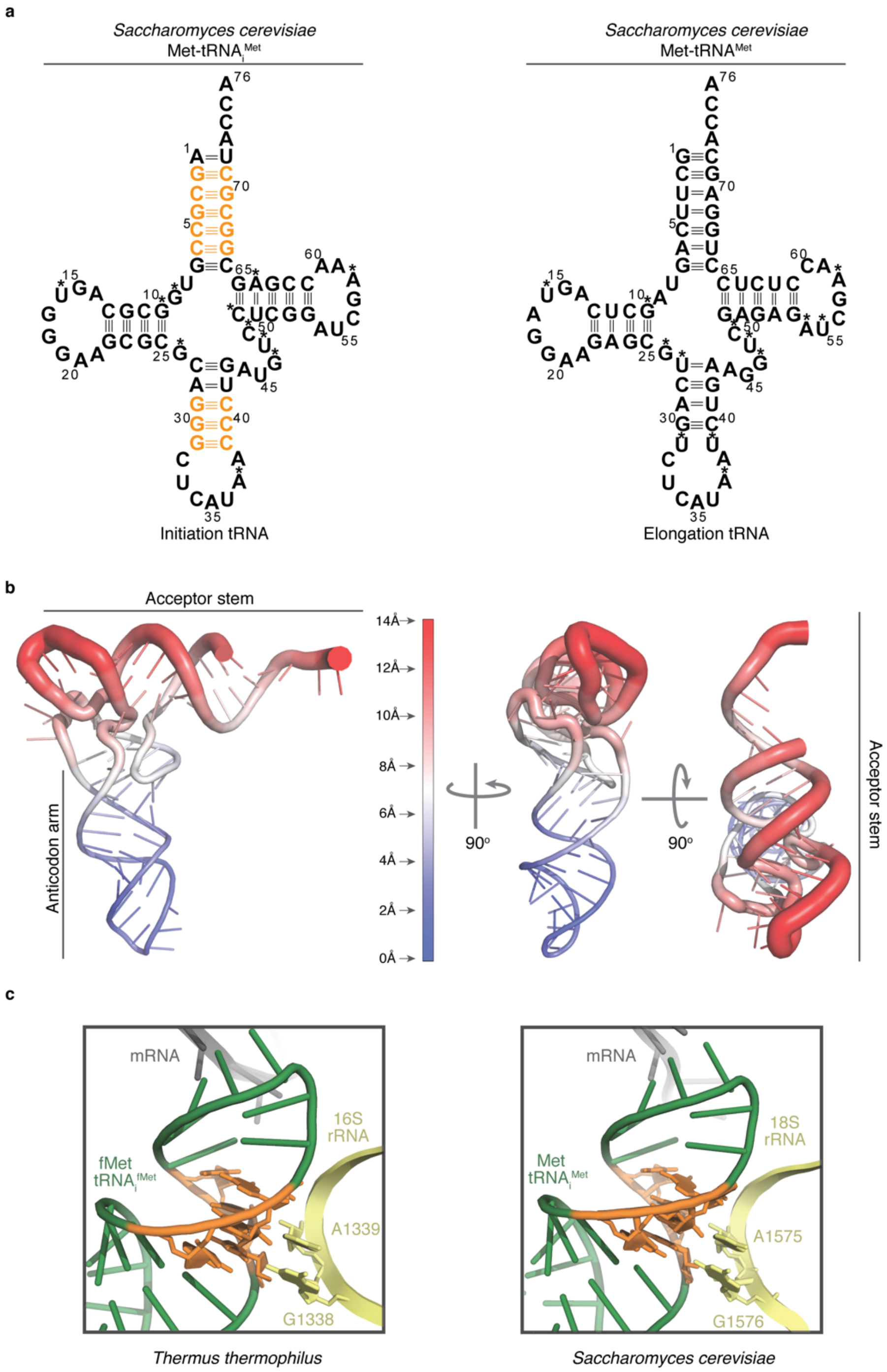

Supplementary Fig. 5. Structural features of the Met-tRNA$_i^{Met}$.
a Left, *S.cerevisiae* sequence for tRNA$_i^{Met}$ with the G-C base pair clusters unique to initiator tRNA in orange. Right, elongation tRNA^{Met} sequence in *S.cerevisiae.* Asterisks denote modified nucleotides. **b** Root mean square deviations (r.m.s.d) between atoms computed for a tRNA in an elongation configuration versus the configuration described in this work. Major differences are found within the acceptor stem. **c** P site interactions established via A minor interactions with ribosomal bases of the small subunits are conserved. Shown on the left is the P site region of a representative bacterial structure (PDB 1vy4) and on the right, that of the current structure.

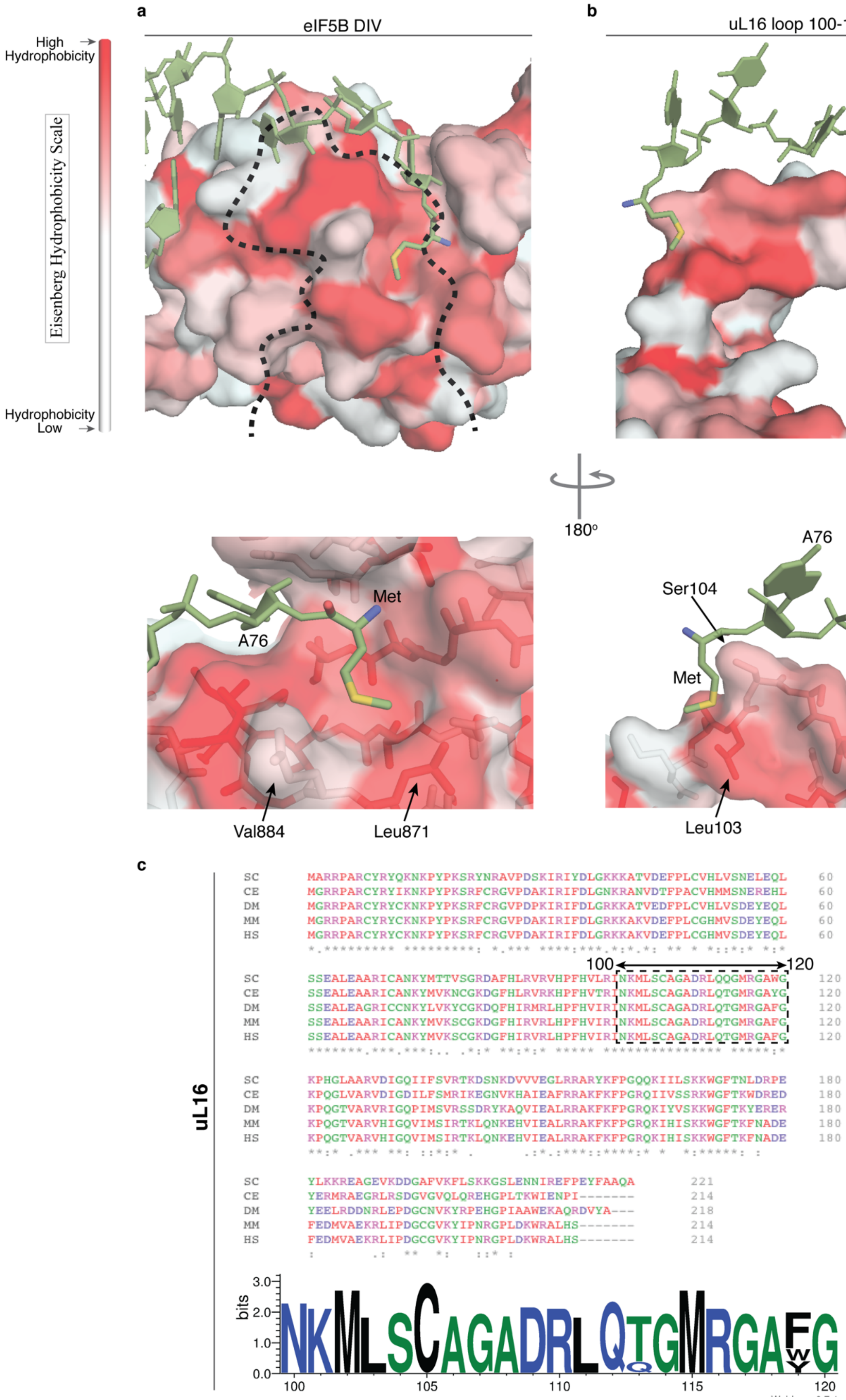

a
eIF5B DIV
b
uL16 loop 100-120
High Hydrophobicity
Eisenberg Hydrophobicity Scale
Hydrophobicity Low
180°
Met
A76
Val884
Leu871
A76
C75
Ser104
Met
Leu103
c
uL16
SC MARRPARCYRYQKNKPYPKSRYNRAVPDSKIRIYDLGKKKATVDEFPLCVHLVSNELEQL 60
CE MGRRPARCYRYIKNKPYPKSRFCRGVPDAKIRIFDLGNKRANVDTFPACVHMMSNEREHL 60
DM MGRRPARCYRYCKNKPYPKSRFCRGVPDPKIRIFDLGRKKATVEDFPLCVHLVSDEYEQL 60
MM MGRRPARCYRYCKNKPYPKSRFCRGVPDAKIRIFDLGRKKAKVDEFPLCGHMVSDEYEQL 60
HS MGRRPARCYRYCKNKPYPKSRFCRGVPDAKIRIFDLGRKKAKVDEFPLCGHMVSDEYEQL 60
100 120
SC SSEALEAARICANKYMTTVSGRDAFHLRVRVHPFHVLRINKMLSCAGADRLQQGMRGAWG 120
CE SSEALEAARICANKYMVKNCGKDGFHLRVRKHPFHVTRINKMLSCAGADRLQTGMRGAYG 120
DM SSEALEAGRICCNKYLVKYCGKDQFHIRMRLHPFHVIRINKMLSCAGADRLQTGMRGAFG 120
MM SSEALEAARICANKYMVKSCGKDGFHIRVRLHPFHVIRINKMLSCAGADRLQTGMRGAFG 120
HS SSEALEAARICANKYMVKSCGKDGFHIRVRLHPFHVIRINKMLSCAGADRLQTGMRGAFG 120
SC KPHGLAARVDIGQIIFSVRTKDSNKDVVVEGLRRARYKFPGQQKIILSKKWGFTNLDRPE 180
CE KPQGLVARVDIGDILFSMRIKEGNVKHAIEAFRRAKFKFPGRQIIVSSRKWGFTKWDRED 180
DM KPQGTVARVRIGQPIMSVRSSDRYKAQVIEALRRAKFKFPGRQKIYVSKKWGFTKYERER 180
MM KPQGTVARVHIGQVIMSIRTKLQNKEHVIEALRRAKFKFPGRQKIHISKKWGFTKFNADE 180
HS KPQGTVARVHIGQVIMSIRTKLQNKEHVIEALRRAKFKFPGRQKIHISKKWGFTKFNADE 180
SC YLKKREAGEVKDDGAFVKFLSKKGSLENNIREFPEYFAAQA 221
CE YERMRAEGRLRSDGVGVQLQREHGPLTKWIENPI------- 214
DM YEELRDDNRLEPDGCNVKYRPEHGPIAAWEKAQRDVYA--- 218
MM FEDMVAEKRLIPDGCGVKYIPNRGPLDKWRALHS------- 214
HS FEDMVAEKRLIPDGCGVKYIPNRGPLDKWRALHS------- 214
bits
3.0
2.0
1.0
0.0
100
105
110
115
120

Supplementary Fig. 6. Surface hydrophobicity calculations for eIF5B and uL16 and sequence conservation across eukaryotes.

a eIF5B domain IV represented as Van der Waals surface colored according to the Eisenberg hydrophobicity scale. Met-tRNA$_i^{Met}$ is shown as sticks. The position occupied by the uL16 loop (residues 100-120) is indicated with a dashed line. **b** Same representation as in **a** from the uL16 view. Methionine residue esterified to the 3'OH of Met-tRNA$_i^{Met}$ A76 is hosted in a highly hydrophobic cavity formed by eIF5B DIV and the uL16 loop (residues 100-120). **c** Sequence alignment of uL16 from *Saccharomyces cerevisiae* (SC), *Caenorhabditis elegans* (CE), *Drosophila melanogaster* (DM), *Mus musculus* (MM) and humans (HS). The residues 100-120 loop is highly conserved across eukaryotes.

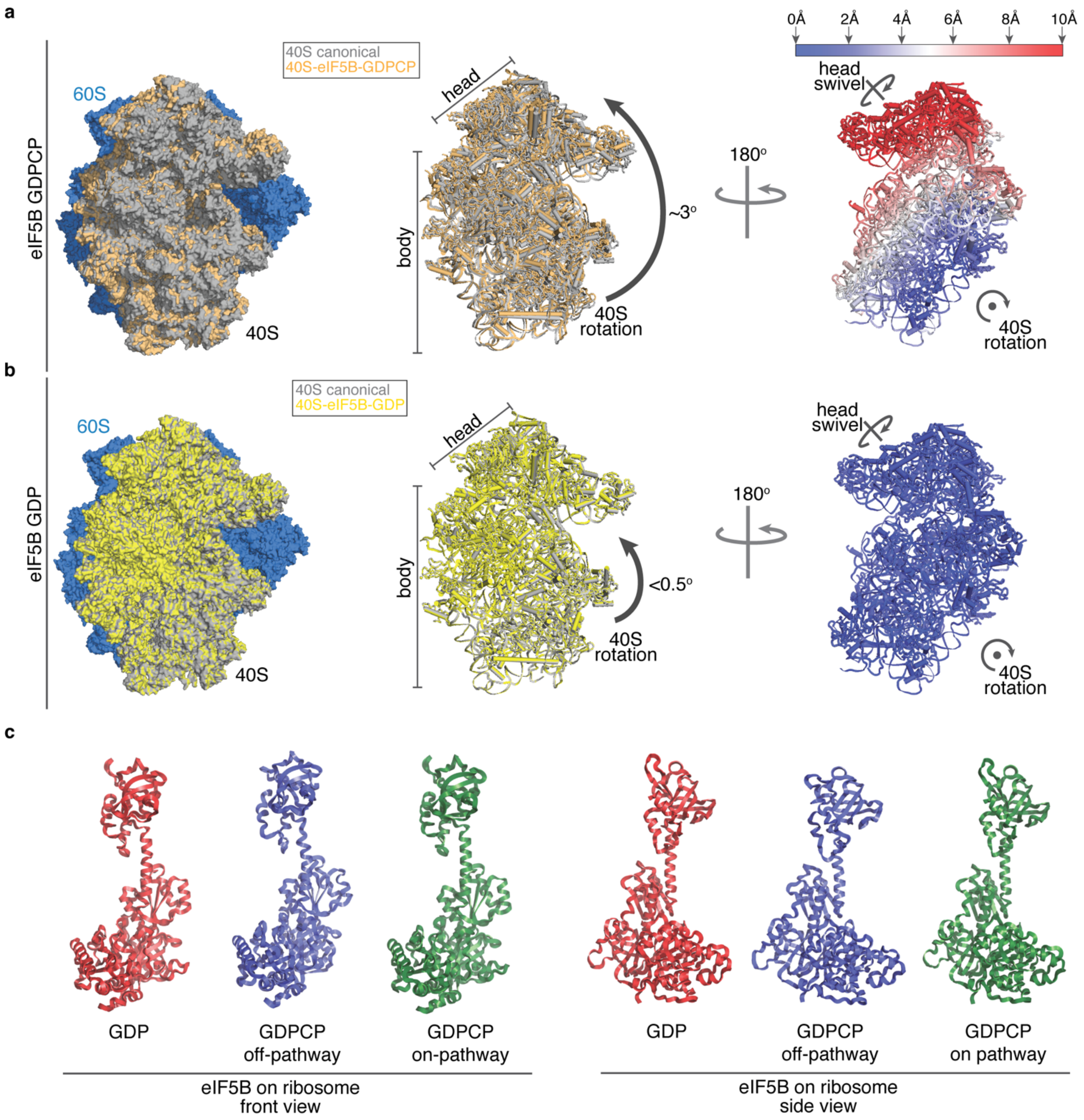

Supplementary Fig. 7. 40S subunit rotation state in eIF5B containing complexes.
a In the presence of a non-hydrolysable GTP analog, binding of eIF5B to the 80S induces a moderate degree of 40S rotation compared with a canonical state[5,6]. **b** In the present reconstruction, with eIF5B in the 80S complex and hydrolyzed GTP, the 40S subunit features an almost canonical configuration with very little rotation and/or head swivel. **c** On-ribosome eIF5B conformations in different nucleotide states.

Supplementary Movie 1. Met-tRNA$_i^{Met}$ transition from late initiation to elongation.

The interaction DIV of eIF5B establishes with the acceptor stem of Met-tRNA$_i^{Met}$ keeps the $_{73}$ACCA$_{76}$-Met of the tRNA away from the PTC (blue). Upon eIF5B departure, the $_{73}$ACCA$_{76}$-Met is free to accommodate into the PTC such that the Met-tRNA$_i^{Met}$ will adopt a fully elongation-competent conformation.

Supplementary Table 1. Cryo-EM data collection, refinement and validation statistics

	EMDB-21859<br>PDB 6WOO
**Data collection and processing**	
Magnification	130,000
Voltage (kV)	300
Electron exposure (e–/Å^2)	49.43
Defocus range (μm)	-0.5/-2
Pixel size (Å)	0.83
Symmetry imposed	C1
Initial particle images (no.)	289,689
Final particle images (no.)	190,058
Map resolution (Å)	2.9
FSC threshold	0.143
Map resolution range (Å)	3-8
**Refinement**	
Initial model used (PDB code)	4V7R
Model resolution (Å)	3.3
FSC threshold	0.5
Model resolution range (Å)	3-8
Map sharpening *B* factor (Å^2)	-78.24
Model composition:	
Non-hydrogen atoms	211,067
Ligands	52
B factors (Å^2):	
Protein	109.4
RNA	101.1
R.m.s. deviations:	
Bond lengths (Å)	0.010
Bond angles (°)	1.43
Validation	
MolProbity score	2.42
Clashscore	3.98
Poor rotamers (%)	6.43
Ramachandran plot:	
Favored (%)	87.77
Allowed (%)	98.13
Disallowed (%)	1.86
RNA validation:	
Angles outliers (%)	0.19
Sugar puckers outliers (%)	2.05
Average suit	0.456

Supplementary Table 2. Source data for Fig. 2h,i

Panel h	GCN4-lacZ					
Biological replicates	WT		eIF5BΔ		R955A	
	R (no 3-AT)	DR (+3-AT)	R (no 3-AT)	DR (+3-AT)	R (no 3-AT)	DR (+3-AT)
1st	13.22	110.49	7.22	9.26	7.95	44.43
	6.54	99.59	2.50	3.05	6.72	36.45
	6.20	106.08	6.79	6.03	7.12	30.83
2nd	10.90	98.10	4.84	3.76	13.10	47.02
	12.24	104.38	6.00	5.90	13.35	39.28
	10.86	92.96	9.41	4.01	10.55	40.04
average	9.99	101.93	6.13	5.34	9.80	39.67
standard deviation	2.94	6.28	2.34	2.26	2.97	5.75

Panel i	Leaky scanning/GCN4-lacZ		
Biological replicates	WT	eIF5BΔ	R955A
	R (no 3-AT)	R (no 3-AT)	R (no 3-AT)
1st	31.35	315.30	192.20
	35.46	185.08	119.06
	54.85	275.40	164.31
2nd	39.58	158.95	205.49
	36.79	210.69	161.83
	40.18	233.26	166.35
average	39.70	229.78	168.21
standard deviation	8.07	57.94	29.80

References

1 Wang, J. *et al.* eIF5B gates the transition from translation initiation to elongation. *Nature* **573**, 605-608, doi:10.1038/s41586-019-1561-0 (2019).

2 Tan, Y. Z. *et al.* Addressing preferred specimen orientation in single-particle cryo-EM through tilting. *Nat Methods* **14**, 793-796, doi:10.1038/nmeth.4347 (2017).

3 Brown, A. *et al.* Tools for macromolecular model building and refinement into electron cryo-microscopy reconstructions. *Acta Crystallogr D Biol Crystallogr* **71**, 136-153, doi:10.1107/S1399004714021683 (2015).

4 Nicholls, R. A., Long, F. & Murshudov, G. N. Low-resolution refinement tools in REFMAC5. *Acta Crystallogr D Biol Crystallogr* **68**, 404-417, doi:10.1107/S090744491105606X (2012).

5 Fernandez, I. S. *et al.* Molecular architecture of a eukaryotic translational initiation complex. *Science* **342**, 1240585, doi:10.1126/science.1240585 (2013).

6 Huang, B. Y. & Fernandez, I. S. Long-range interdomain communications in eIF5B regulate GTP hydrolysis and translation initiation. *Proc Natl Acad Sci U S A* **117**, 1429-1437, doi:10.1073/pnas.1916436117 (2020).
