# Supplementary material for: Structural basis for the transition from translation initiation to elongation by an 80S-eIF5B complex
Source: Nat Commun. 2020 Oct 6;11:5003. doi: 10.1038/s41467-020-18829-3 (PMC7538418; doi:10.1038/s41467-020-18829-3)
Supplement: Supplementary file 3 — Description of Additional Supplementary Files [file 41467_2020_18829_MOESM3_ESM.pdf]

Description of Additional Supplementary Files

File name: Supplementary Movie 1
Description: Met-tRNAi^{Met} transition from late initiation to elongation.The interaction DIV of eIF5B establishes with the acceptor stem of Met-tRNAi^{Met} keeps the 73ACCA76-Met of the tRNA away from the PTC (blue). Upon eIF5B departure, the 73ACCA76-Met is free to accommodate into the PTC such that the Met-tRNAi^{Met} will adopt a fully elongation-competent conformation.
